# Curtain grouting volume prediction using a Bayesian-optimized stacking ensemble model with SHAP analysis

**DOI:** 10.1038/s41598-026-45538-6

**Published:** 2026-04-01

**Authors:** Yahui Ma, Zhanquan Yuan, Bo Xiong, Hongwei Lei, Weiquan Zhao

**Affiliations:** 1 State Key Laboratory of Simulation and Regulation of Water Cycle in River Basin, China Institute of Water Resources and Hydropower Research, Beijing, 100038 China; 2 China Gezhouba Group Co., Ltd, Yichang, 443000 China; 3 China Gezhouba Group Municipal Engineering Co., Ltd, Yichang, 443000 China

**Keywords:** Curtain grouting, Grouting volume prediction, Stacking ensemble, Bayesian optimization, SHAP, Energy science and technology, Engineering, Solid Earth sciences

## Abstract

Curtain grouting is widely used to control seepage in large-scale water conservancy and hydropower projects, and accurate prediction of grouting volume is essential for ensuring construction quality and cost control. This study proposes a grouting volume prediction model combining Bayesian optimization (BO) with a stacking ensemble learning framework. The model was developed using a dataset of 778 valid grouting records and incorporated seven key input features: hole sequence, hole depth, section length, hole diameter, pre-grouting permeability, initial water-cement ratio, and grouting pressure. Within this framework, XGBoost, LightGBM, and Random Forest were employed as base learners, with BO applied for global hyperparameter optimization. Ridge regression served as the meta-learner to construct the Bayesian-optimized stacking ensemble (BO-Stacking) model. SHapley Additive exPlanations (SHAP) analysis was used to quantify feature contributions and enhance model interpretability. The results show that the BO-Stacking model outperformed the benchmark models, achieving a coefficient of determination (*R*^2^) of 0.92, a mean absolute error (MAE) of 70.19 L, and a root mean square error (RMSE) of 187.07 L. Scatter analysis further indicated strong agreement between predicted and measured values. SHAP analysis quantified the relative contributions of geological conditions, construction parameters, and slurry properties to grouting volume. Overall, the proposed approach improves predictive performance of grouting volume under complex geological conditions and provides support for construction planning and quality management.

## Introduction

Grouting is a critical seepage-control technique widely used in major infrastructure projects, including hydraulic and hydropower engineering, transportation tunnels, and mining engineering^1^. Its primary objective is to form continuous and dense seepage curtains or reinforcement zones within rock and soil masses, thereby blocking groundwater flow paths and improving foundation bearing capacity^2^. In practice, grouting is a concealed process characterized by substantial uncertainty. Its effectiveness is governed by the coupled effects of geological conditions, grout rheology, and construction parameters^3^. Complex subsurface conditions further increase the uncertainty of grouting performance and the difficulty of construction control^4,5^. Under these conditions, accurate prediction of grouting volume has become a key scientific and engineering challenge for design optimization, refined construction control, and quality assessment. Recent studies in geotechnical and underground engineering have focused on slope stability, rock fracture evolution, and anchorage mechanisms under complex geological conditions^6–9^. However, quantitative prediction of grouting volume in curtain grouting remains insufficiently explored relative to these well-studied topics,

Early approaches to grouting volume prediction primarily relied on empirical relationships, engineering analogy, and numerical simulation. Empirical methods estimate grouting volume by combining simplified theoretical assumptions with historical experience and project-specific parameters^10^, whereas analogy-based methods extrapolate predictions from comparable projects^11^. Numerical simulation approaches attempt to reproduce grout migration behavior using mechanistic models under prescribed boundary conditions^12^. Although these methods have contributed to engineering practice, they are often limited in predictive accuracy and transferability under complex field conditions.

Unlike conventional model-driven approaches that rely on explicit governing equations, machine learning methods learn relationships directly from historical data, thereby capturing complex nonlinear patterns and interactions among high-dimensional variables^13–18^. With advances in artificial intelligence, big data technology, and engineering monitoring systems^19^, machine learning has emerged as a promising data-driven approach for grouting volume prediction^20–24^. However, the predictive performance of a single model is typically constrained by algorithmic bias and sensitivity to data heterogeneity. In practice, grouting datasets often vary across sites, strata, and construction stages, and measurement noise and response imbalance are common. Consequently, single models may exhibit unstable performance and limited generalizability when applied to conditions beyond those represented in the training data.

To address these limitations, ensemble learning has attracted increasing attention as an effective strategy for improving predictive accuracy and stability in engineering applications^25–27^. Representative ensemble paradigms include bagging, boosting, and stacking^28^. Bagging reduces variance primarily through bootstrap sampling and parallel training, whereas boosting enhances accuracy by iteratively correcting prediction errors and thereby reducing bias. In contrast, stacking improves predictive performance by integrating predictions from multiple base learners. Its core mechanism involves leveraging a meta-learner to combine the outputs of base learners, thereby integrating complementary model strengths and improving overall predictive accuracy and generalization^29^.

In constructing the stacking model, base learners were selected according to the principles of both accuracy and diversity. Specifically, XGBoost^30^ achieves strong predictive accuracy and generalization through regularization and precise loss approximation, whereas LightGBM^31^ demonstrates high training efficiency and low memory consumption in large-scale and high-dimensional settings. Random Forest (RF)^32^, as a representing bagging-based algorithm, is highly robust to noise and effective in reducing variance. These three algorithms process data through distinct mechanisms, thereby meeting the fundamental requirements for base learners. Because base-learner predictions often exhibit high correlation, which may lead to multicollinearity, ridge regression was selected as the meta-learner. By introducing an L2 regularization penalty, ridge regression suppresses coefficient instability and enhances the stability of the final prediction^33^. Accordingly, this study used XGBoost, LightGBM, and RF as base learners, with ridge regression as the meta-learner to construct the stacking ensemble model for grouting volume prediction.

Because ensemble models involve numerous hyperparameters and their performance is highly sensitive to parameter configuration, empirical tuning is often insufficient to identify optimal solutions^34^. Although grid search (GS) and random search (RS)^35^ are commonly used for hyperparameter tuning, both methods show clear limitations when applied to high-dimensional parameter spaces. In contrast, Bayesian optimization (BO), as an efficient global optimization strategy, employs surrogate models to approximate the objective function based on historical evaluations, thereby guiding the search process more precisely and converging to the global optimum^36^. Compared with metaheuristic algorithms such as particle swarm optimization (PSO)^37^ and grey wolf optimizer (GWO)^38^, BO offers advantages in search efficiency while also demonstrating greater capacity to handle noise and uncertainty. Therefore, this study employed BO for hyperparameter optimization to improve model predictive performance under a limited computational budget.

Although ensemble learning methods can improve predictive accuracy, their complex internal structures often reduce model interpretability^39^. To address this limitation, explainable artificial intelligence (XAI) has emerged as an important research direction in intelligent engineering. Among the available techniques, SHapley Additive exPlanations (SHAP), grounded in cooperative game theory, provides an effective means of quantifying feature contributions to model predictions, owing to its sound theoretical foundation and consistent explanatory framework^40^. SHAP quantify the marginal contribution of each input feature to the model output and provide both global feature-importance rankings and local prediction explanations, thereby helping to interpret the nonlinear and interactive patterns learned by the model. Although some studies have applied SHAP in civil engineering^41–43^, research on model interpretability for curtain grouting volume prediction remains limited.

**Fig. 1:**
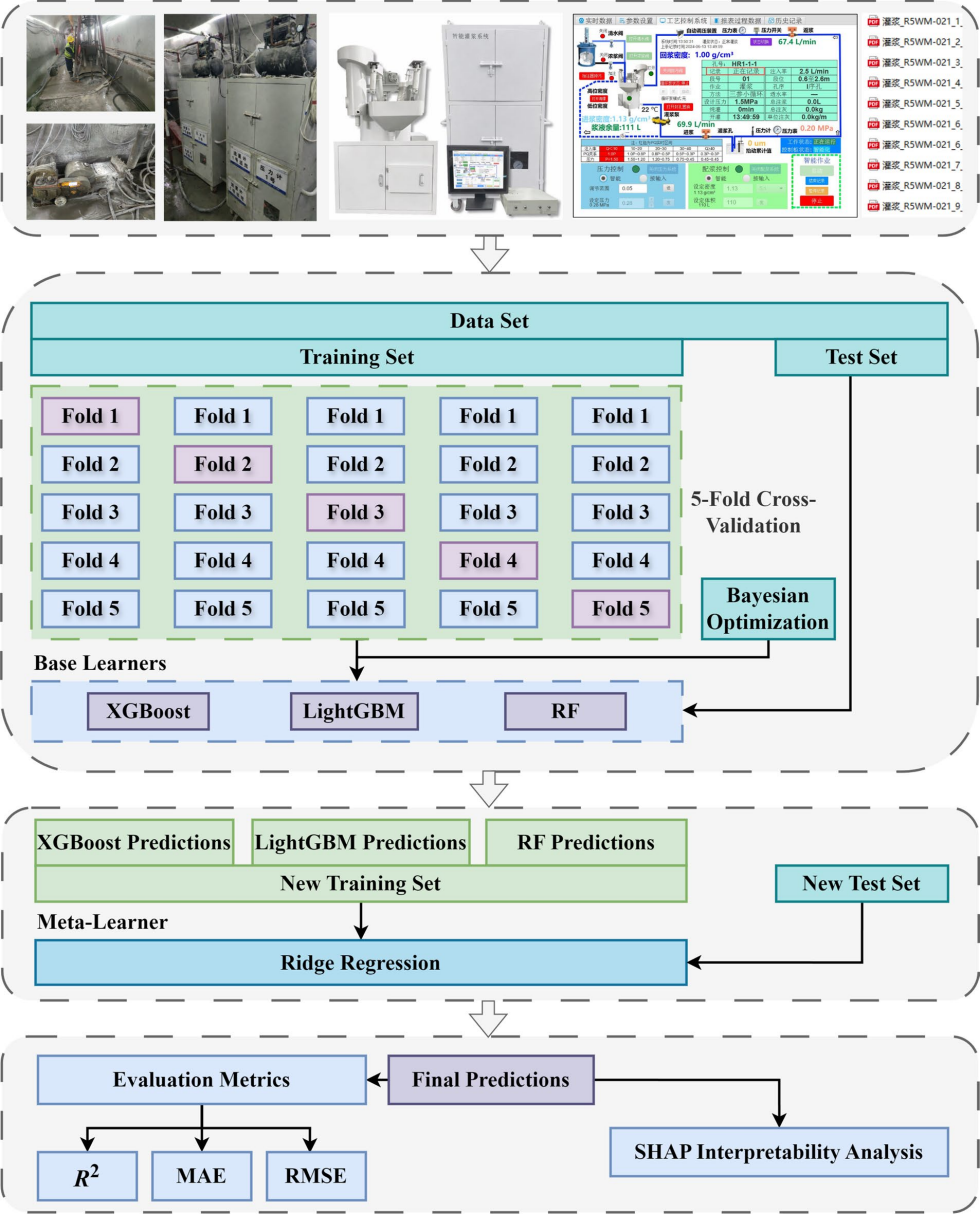
Research framework.

To address the challenges outlined above, this study develops a curtain grouting volume prediction model based on a Bayesian-optimized stacking ensemble (BO-Stacking) model with SHAP-based interpretability analysis. The model was validated using field data collected from a large-scale water conservancy project in Xinjiang via an intelligent grouting monitoring system. The overall workflow is illustrated in Fig. 1 and consists of three main steps: feature selection and data acquisition, construction of the BO-Stacking model, and model evaluation with SHAP analysis.

## Research framework

Using engineering data from a large-scale water conservancy project in Xinjiang, this study constructed and validated a BO-Stacking model for curtain grouting volume prediction. The technical framework is illustrated in Fig. 1, and the specific implementation steps are detailed as follows:

(1) Feature selection and data acquisition. The primary factors influencing grouting volume include geological attributes, construction parameters, and slurry properties^25^. Based on grouting mechanisms and engineering experience, seven key parameters were selected as input features: hole sequence, hole depth, section length, hole diameter, pre-grouting permeability (Lugeon), initial water–cement ratio, and grouting pressure. Grouting volume served as the target variable. The data were obtained from real-time monitoring records of the project’s intelligent grouting system. The dataset covered the period from April to July 2025 and comprised 778 valid grouting records across five curtain grouting units. These five units encompassed distinct construction zones and local geological conditions within the project, thereby improving the diversity of the dataset. Table 1 presents the descriptive statistics of the feature parameters in the raw dataset. Following preprocessing, the dataset was divided into training and test sets at an 8:2 ratio for model construction and performance evaluation.

(2) Construction of the BO-Stacking model. XGBoost, LightGBM, and RF were used as base learners. To mitigate the risk of overfitting, the base learners were trained using five–fold cross-validation. Ridge regression was served as the meta-learner to integrate base-learner predictions. The out-of-fold predictions generated during five-fold cross-validation were used as inputs to train the meta-learner, thereby preventing data leakage in the stacking procedure. The BO algorithm was applied to optimize hyperparameters of all base learners.

(3) Model evaluation with SHAP analysis. To evaluate the predictive performance of the proposed BO-Stacking model, its predictive results on the test set were compared against those of the benchmark models. The coefficient of determination (*R*^2^), mean absolute error (MAE), and root mean square error (RMSE) were selected as evaluation metrics to quantify predictive performance across multiple dimensions. Furthermore, SHAP analysis was incorporated to enhance model interpretability by quantifying the marginal contributions of each input features to grouting volume.

**Table 1:** Descriptive statistics of parameters.

Parameter	Unit	Count	Mean	Std	Min	25%	50%	75%	Max
Hole sequence	-	778	2.26	0.81	1.00	2.00	2.00	3.00	3.00
Hole depth	m	778	25.29	15.32	2.41	10.41	25.41	38.90	55.41
Section length	m	778	4.56	0.98	2.00	5.00	5.00	5.00	5.00
Hole diameter	mm	778	58.16	6.21	56.00	56.00	56.00	56.00	76.00
Pre-grouting permeability	Lu	778	3.05	10.57	0.00	0.10	0.58	2.92	106.58
Initial water-cement ratio	-	778	4.96	0.31	1.00	5.00	5.00	5.00	5.00
Grouting pressure	MPa	778	4.02	0.94	1.50	3.50	4.50	4.97	5.09
Grouting volume	L	778	275.69	687.11	0.00	60.12	137.80	536.00	5155.60

## Methodology

### Stacking ensemble

**Fig. 2:**
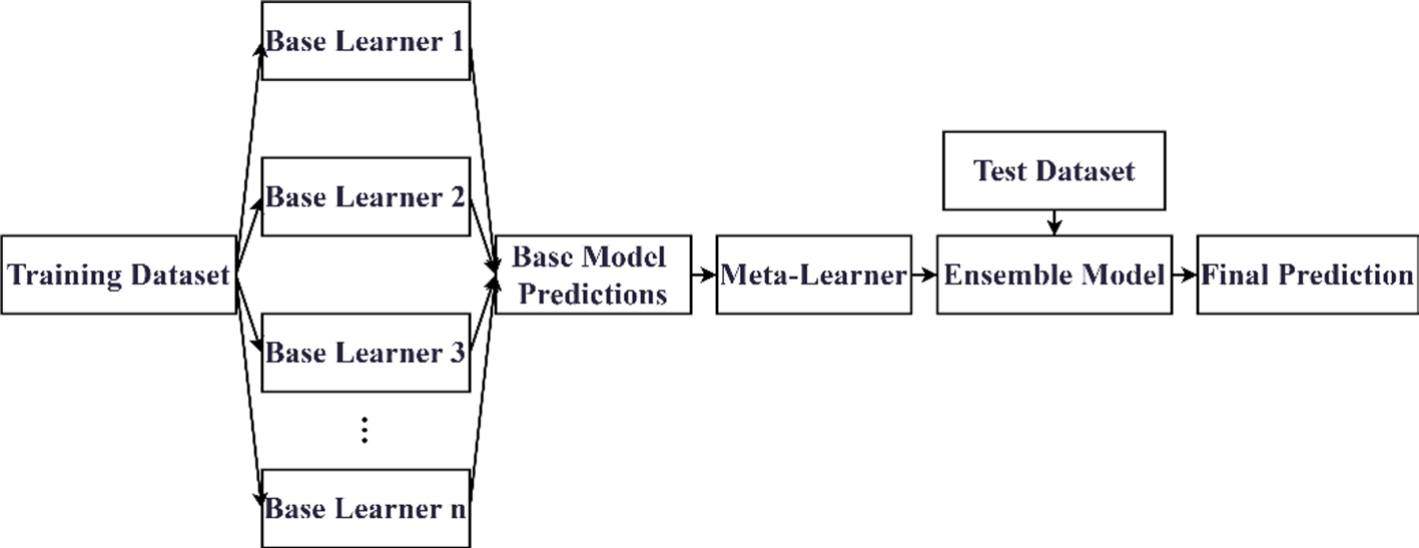
Stacking ensemble learning framework.

Stacking is an ensemble learning strategy designed to enhance model generalizability. Its core mechanism is to construct a multilevel prediction structure that leverages the complementary strengths of different base models in data representation and pattern recognition^44^. In the first layer, multiple base learners are trained independently and generate predictions on the training data. In the second layer, a meta-learner is trained to combine the predictions of the base learners through learned weighting or nonlinear mapping. By compensating for the biases of the base learners, the meta-learner produces a more accurate and robust final prediction. The overall architecture of the stacking ensemble is illustrated in Fig. 2.

### Base learners

#### XGBoost

XGBoost is an ensemble learning framework widely applied in various predictive modeling tasks. Its core mechanism is based on an additive model and a forward stagewise optimization strategy, in which decision trees are iteratively constructed to correct the residuals of the preceding iteration^30^. Unlike conventional gradient boosting methods, the objective function of XGBoost considers not only training loss term but also a regularization term to balance predictive accuracy and model complexity. The regularization term penalizes model complexity, thereby mitigating overfitting and improving generalization. The objective function is given by Eqs. (1) and (2).

1 $$\:Obj={\sum\:}_{i=1}^{n}l({y}_{i},{\widehat{y}}_{i})+{\sum\:}_{k=1}^{K}{\Omega\:}\left({f}_{k}\right)$$

2 $$\:{\Omega\:}\left({f}_{k}\right)=\gamma\:T+\frac{1}{2}\lambda\:{\sum\:}_{j=1}^{T}({\omega\:}_{j}{)}^{2}$$

where *Obj* is the objective function; *l* is the loss function measuring the discrepancy between the predicted $\:{\widehat{y}}_{i}$ and actual values $\:{y}_{i}$; $\:{f}_{k}$ is the *k*-th decision tree; *K* is the total number of decision trees; $\:{\Omega\:}\left({f}_{k}\right)$ is the regularization term of the *k*-th tree; $\:\gamma\:$ and $\:\lambda\:$ are regularization hyperparameters; *T* is the total number of leaf nodes; and $\:{\omega\:}_{j}$ is the weight of the *j*-th leaf node.

#### LightGBM

LightGBM is a distributed ensemble learning framework designed for handling large-scale data and high-dimensional features. Its core mechanism lies in the adoption of two algorithmic techniques: Gradient-based One-Side Sampling (GOSS) and Exclusive Feature Bundling (EFB)^31^. The overall prediction of LightGBM is obtained by accumulating the outputs of all constituent trees, as given by Eq. (3).

3 $$\:{F}_{m}\left(x\right)={F}_{m-1}\left(x\right)+{\rho\:}_{m}{h}_{m}\left(x\right)$$

where $\:{F}_{m}\left(x\right)$ is the final prediction; $\:{F}_{m-1}\left(x\right)$ is the cumulative prediction of the previous *m* − 1 trees; $\:{h}_{m}\left(x\right)$ is the prediction result of the *m*-th tree for input *x*; and $\:{\rho\:}_{m}$ is the learning rate or weight assigned of the *m*-th tree.

#### Random forest

Random Forest is a parallel ensemble learning algorithm based on the bagging methodology^32^. It constructs and aggregates multiple independent decision trees to achieve robust predictive performance. First, bootstrap sampling with replacement is applied to the original training set to generate independent training subsets for each decision tree. Second, at each node during tree construction, a random subset of features is selected as candidates, and the optimal split feature is identified from this subset. Finally, for regression tasks, the final prediction is obtained by averaging the outputs of all decision trees. The model formulation for regression is given by Eq. (4).

4 $$\:F\left(x\right)=\frac{1}{n}{\sum\:}_{i=1}^{n}{h}_{i}\left(x\right)\:$$

where *n* is the number of decision trees; $\:{h}_{i}\left(x\right)$ is the prediction of the *i*-th tree for input *x*; and $\:F\left(x\right)$ is the final prediction of the RF model.

Since XGBoost, LightGBM, and RF are all tree-based ensemble algorithms that partition the feature space based on rank ordering rather than absolute feature magnitudes, no feature normalization was required prior to model training^45^. These models are inherently invariant to feature scaling and monotonic transformations.

### Meta-learner

As a regularized least squares method designed to address multicollinearity, ridge regression offers notable robustness when employed as a meta-learner. Its core mechanism involves introducing an L2 regularization term into the ordinary least squares loss function to constrain the magnitude of model coefficients, thereby achieving a controlled trade-off between bias and variance. Rather than minimizing the residual sum of squares (RSS) alone, the objective function simultaneously penalizes the magnitude of the regression coefficients, thereby incorporating structural risk into the optimization. The resulting loss function, comprising the RSS and the L2 regularization term, is given by Eq. (5).

5 $$\:L\left(\theta\:\right)={\sum\:}_{i=1}^{n}({y}_{i}-{\sum\:}_{j=1}^{m}{\theta\:}_{j}{x}_{ij}{)}^{2}+\lambda\:{\sum\:}_{j=1}^{m}{\theta\:}_{j}^{2}$$

where *n* is the number of samples; *m* is the number of features; $\:{y}_{i}$ is the target value of the *i*-th sample; $\:{x}_{ij}$ is the *j*-th feature value of the *i*-th sample; $\:{\theta\:}_{j}$ is the weight of the *j*-th feature; and $\:\lambda\:$ is the regularization parameter controlling the strength of the penalty.

Because ridge regression is sensitive to feature scale, the meta-features were standardized using Z-score normalization prior to meta-learner training. The scaler was fitted exclusively on the training meta-features and subsequently was applied to the validation and test meta-features to prevent data leakage.

### Bayesian optimization

**Fig. 3:**
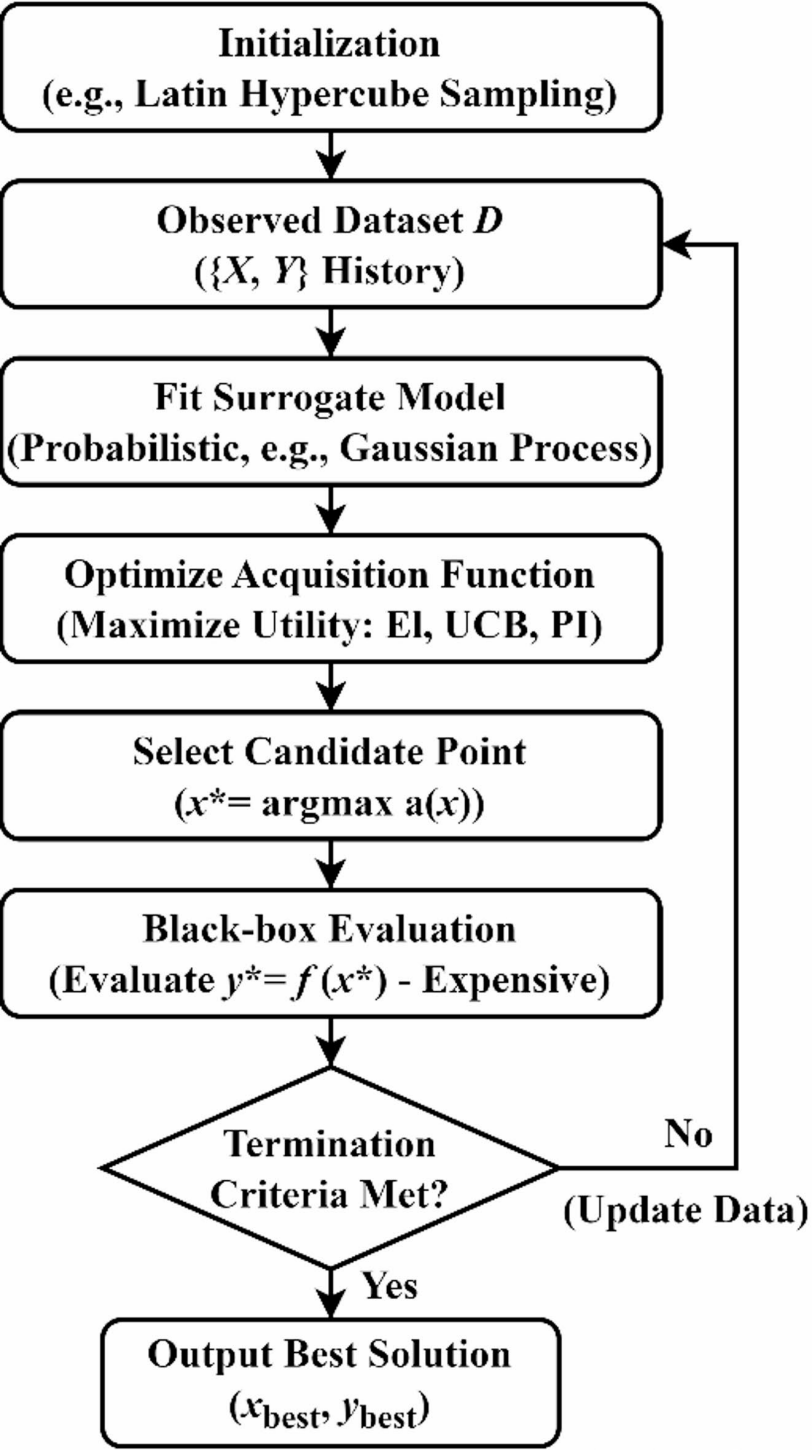
Flowchart of the BO algorithm.

BO is a sequential global optimization method based on probabilistic surrogate models. It is particularly suited for black-box optimization problems in which the objective function is computationally expensive, non-differentiable, or stochastic. Its core mechanism involves employing a Gaussian process (GP) to construct a probabilistic surrogate model of the objective function. By optimizing an acquisition function, the algorithm balances exploration and exploitation, thereby converging toward the global optimum within a limited evaluation budget. The overall optimization workflow is illustrated in Fig. 3.

The BO algorithm operates as a closed-loop sequential optimization procedure. In the initialization phase, methods such as Latin hypercube sampling are used to generate an initial sample set, which is subsequently used to train the GP surrogate model and establish a prior approximation of the objective function. The algorithm then enters the iterative phase. In each iteration, the next candidate point is selected by maximizing the acquisition function based on the posterior distribution of the current GP model. The objective function is then evaluated at this candidate point, and the resulting observation is added to the dataset. The GP surrogate model is subsequently refitted using the updated dataset to refine the posterior approximation of the objective function. This procedure is repeated until a predefined convergence criterion or computational budget is reached, at which point the optimal hyperparameter configuration is returned.

### SHAP

SHAP is a feature attribution method based on cooperative game theory, designed to provide consistent and interpretable explanations for the predictions of complex models. Its theoretical foundation derives from the Shapley value concept in cooperative game theory, which treats model prediction as a cooperative game^46^, which all features contribute jointly to the output. The contribution of each feature is quantified as the weighted average of its marginal contributions across all possible feature coalition orderings.

The Shapley attribution value of feature *i* for sample *x* quantifies its marginal contribution to the model output. Based on this attribution mechanism, SHAP decomposes the model output into a base value and the sum of individual feature contributions, thereby forming an additive explanation model. The mathematical formulations are given by Eqs. (6) and (7).

6 $$\:{\varphi\:}_{i}\left(x\right)={\sum\:}_{S\subseteq\:N\backslash\:\left\{i\right\}}\frac{\left|S\right|!\left(M-\left|S\right|-1\right)!}{M!}\left[{f}_{S\cup\:\left\{i\right\}}\left(x\right)-{f}_{S}\left(x\right)\right]$$

7 $$\:f\left(x\right)={\varphi\:}_{0}+{\sum\:}_{i=1}^{M}{\varphi\:}_{i}$$

where *N* is the set of all features; *S* is a subset excluding feature *i*; |*S*| is the cardinality of subset *S*; *M* is the total number of features; $\:{f}_{S}\left(x\right)$ is the model prediction based on feature subset *S*; $\:{f}_{S\cup\:\left\{i\right\}}\left(x\right)$ is the model prediction after adding feature *i* to subset *S*; $\:f\left(x\right)$ is the model prediction for sample *x*; and $\:{\varphi\:}_{0}$ is the base value.

### Performance evaluation

To evaluate the predictive performance of the proposed model, the coefficient of determination (*R*^2^), mean absolute error (MAE), and root mean square error (RMSE) were selected as evaluation metrics^47,48^. *R*^2^ measures the extent to which the model explains the total variance in the observed data; values closer to 1 indicates better model fit. MAE quantifies the average absolute deviation between predicted and observed values. RMSE reflects the root mean square deviation and assigns greater weight to larger errors. Smaller values of both MAE and RMSE indicate lower prediction error and higher predictive accuracy. The corresponding calculation formulas are given by Eqs. (8)– (10).

8 $$\:{R}^{2}=1-\frac{{\sum\:}_{i=1}^{n}({\widehat{y}}_{i}-{y}_{i}{)}^{2}}{{\sum\:}_{i=1}^{n}(\overline{y}-{y}_{i}{)}^{2}}$$

9 $$\:RMSE=\sqrt{\frac{1}{n}{\sum\:}_{i=1}^{n}({y}_{i}-{\widehat{y}}_{i}{)}^{2}}$$

10 $$\:MAE=\frac{1}{n}{\sum\:}_{i=1}^{n}|{y}_{i}-{\widehat{y}}_{i}|$$

where *n* is the number of samples; $\:{\widehat{y}}_{i}\:$and$\:\:{y}_{i}\:$are the predicted and observed values of the *i*-th sample, respectively; and$\overline{y}$is the mean of the observed values.

## Results

### Feature correlation analysis

**Fig. 4:**
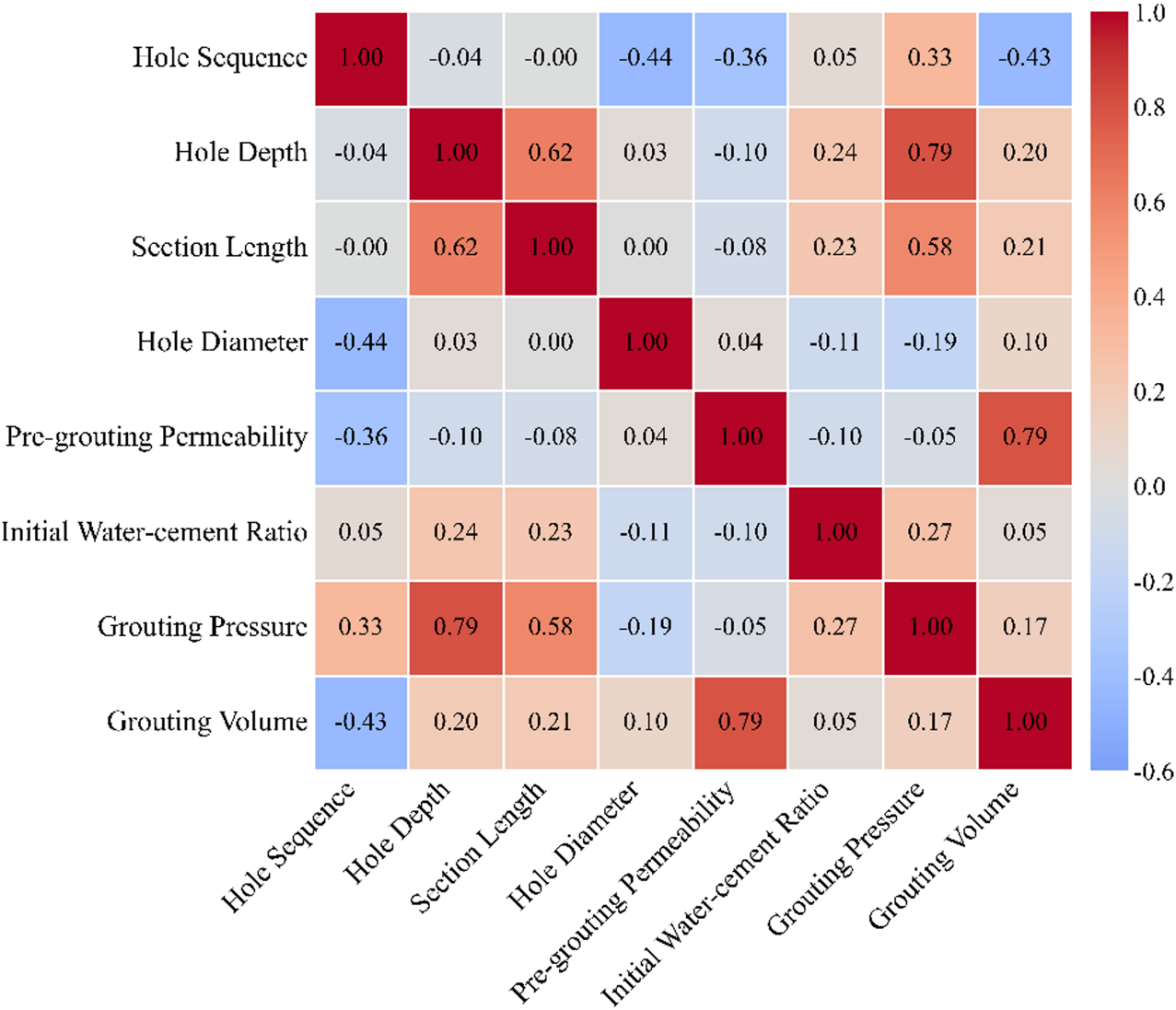
Feature Correlation matrix.

The Pearson correlation coefficient^49^ was used to examine the linear relationships among the input features and the target variable, as illustrated in Fig. 4. In the figure, red and blue indicate positive and negative correlations, respectively, and color intensity reflects the magnitude of the correlation coefficient. The analysis focused on the pairwise correlations between the input features and grouting volume to provide a preliminary assessment of each feature’s linear association with the target variable. As shown in the heatmap, pre-grouting permeability exhibited the strongest positive correlation with grouting volume, with a Pearson coefficient of 0.79. Hole sequence showed a moderate negative correlation with grouting volume, with a Pearson coefficient of − 0.43. Section length and hole depth showed weak to moderate positive correlations with grouting volume, with Pearson coefficients of 0.21 and 0.20, respectively. Grouting pressure and hole diameter showed weaker positive correlations with grouting volume, with Pearson coefficients of 0.17 and 0.10, respectively. The initial water–cement ratio showed the weakest linear correlation with grouting volume, with a Pearson coefficient of only 0.05, suggesting a limited linear association under the current dataset. It should be noted that the Pearson correlation coefficient quantifies only the degree of linear association between variables. It is sensitive to outliers and cannot capture nonlinear relationships or multivariate interaction effects. Therefore, the correlation analysis presented here served primarily as a preliminary quantitative reference for variable relationships. Feature importance and complex interaction effects should be further elucidated through more comprehensive analysis.

### Model training

Following model construction, systematic hyperparameter tuning was conducted to mitigate the risk of overfitting and enhance model generalization performance. Given the complexity of the hyperparameter space, BO was adopted as the primary optimization strategy. The key hyperparameters and their corresponding search ranges for each base learner are summarized in Table 2. All hyperparameters not listed in Table 2 were retained at their default values.

**Table 2:** Hyperparameter configuration of the model.

Model	Hyperparameters	Value	Search range
XGBoost	n_estimators	200	[150, 350]
	learning_rate	0.05	[0.03, 0.15]
	max_depth	4	[3, 6]
	subsample	0.8	[0.65, 0.85]
	colsample_bytree	0.8	[0.65, 0.85]
LightGBM	n_estimators	200	[150, 350]
	learning_rate	0.05	[0.03, 0.15]
	num_leaves	31	[15, 40]
	subsample	0.8	[0.65, 0.85]
	max_depth	6	[3, 7]
RF	n_estimators	300	[100, 400]
	max_depth	10	[6, 15]
	max_features	0.7	[0.5, 0.8]
	min_samples_split	4	[3, 10]

### Model performance evaluation

**Fig. 5:**
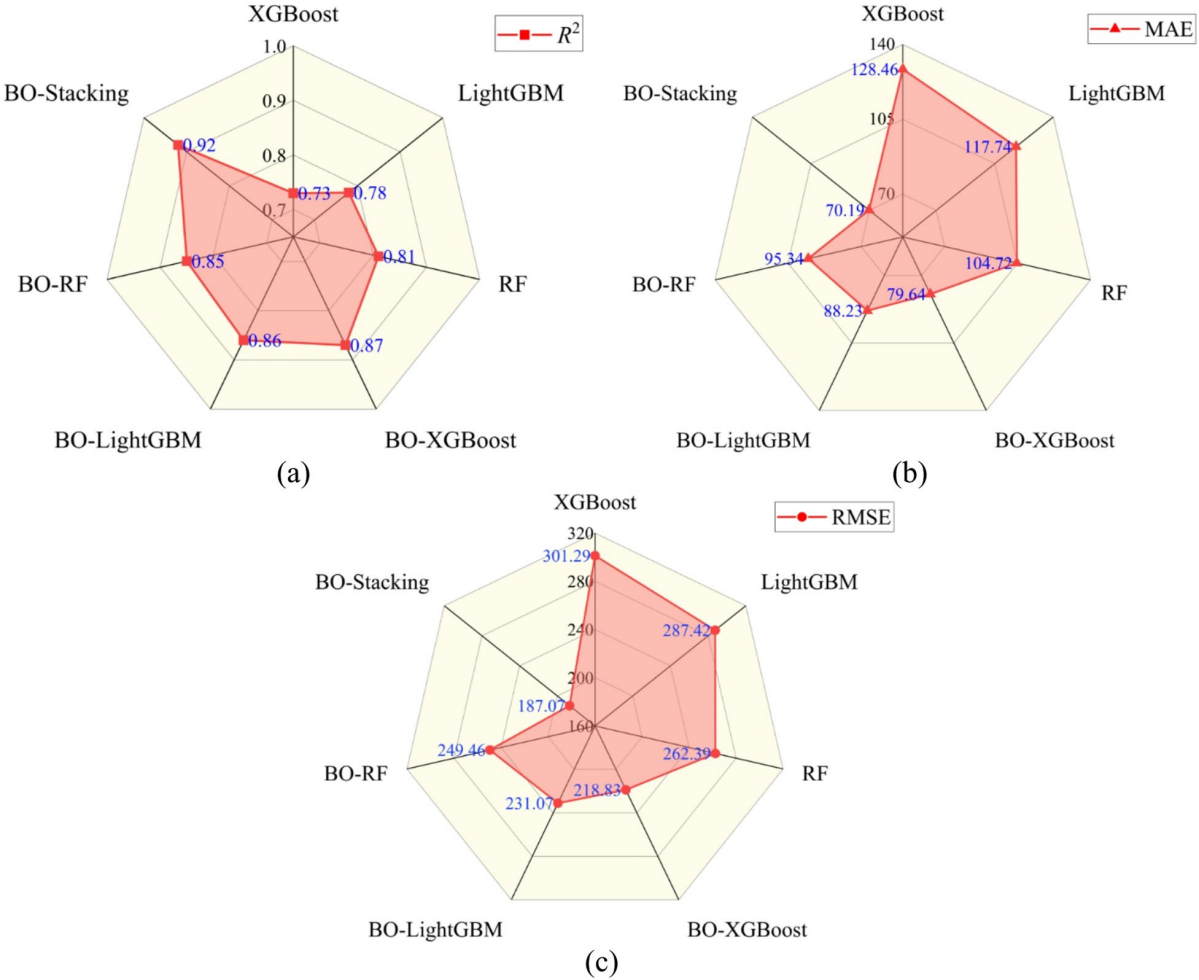
Radar plots of the predictive performance of the seven models: (**a**) *R*^2^; (**b**) MAE; and (**c**) RMSE.

Table 3; Fig. 5 present a comparison of evaluation metrics of the seven models on the test set. In terms of overall performance, the three BO-optimized models showed clear improvements across all metrics relative to their corresponding baseline models. Compared with their unoptimized models, the *R*^2^ values for BO-XGBoost, BO-LightGBM, and BO-RF increased by 19.18%, 10.26%, and 4.94%, respectively; MAE values decreased by 38.00%, 25.06%, and 8.96%, respectively; and RMSE values decreased by 27.37%, 19.61%, and 4.93%, respectively. These results suggested that systematic hyperparameter search via BO yielded parameter configurations more closely aligned with the data distribution. Consequently, the optimized models demonstrated improved ability to capture variations in grouting volume, thereby reducing overall prediction error on the test set.

Building on these results, the BO-Stacking model achieved better predictive performance than the three BO-optimized base learners under the current evaluation setting. Compared with BO-XGBoost, BO-LightGBM, and BO-RF, the *R*^2^ values of BO-Stacking increased by 5.75%, 6.98%, and 8.24%, respectively; MAE values decreased by 11.87%, 20.45%, and 26.38%, respectively; and RMSE values decreased by 14.51%, 19.04%, and 25.01%, respectively. These results suggested that a single learner might be insufficient to fully capture the complex nonlinear relationships between grouting volume and its multiple influencing factors. In contrast, the stacking framework used the predictions of the three BO-optimized base learners as meta-features, enabling the meta-learner to integrate the complementary strengths of different algorithms in characterizing the feature space and error distribution. Consequently, the BO-Stacking model improved the predictive performance relative to the individual base learners.

**Table 3:** Evaluation table of the predictive performance of the models.

Model	*R* ^2^	MAE (L)	RMSE (L)
XGBoost	0.73	128.46	301.29
LightGBM	0.78	117.74	287.42
RF	0.81	104.72	262.39
BO-XGBoost	0.87	79.64	218.83
BO-LightGBM	0.86	88.23	231.07
BO-RF	0.85	95.34	249.46
BO-Stacking	0.92	70.19	187.07

### Visual assessment of prediction accuracy

**Fig. 6:**
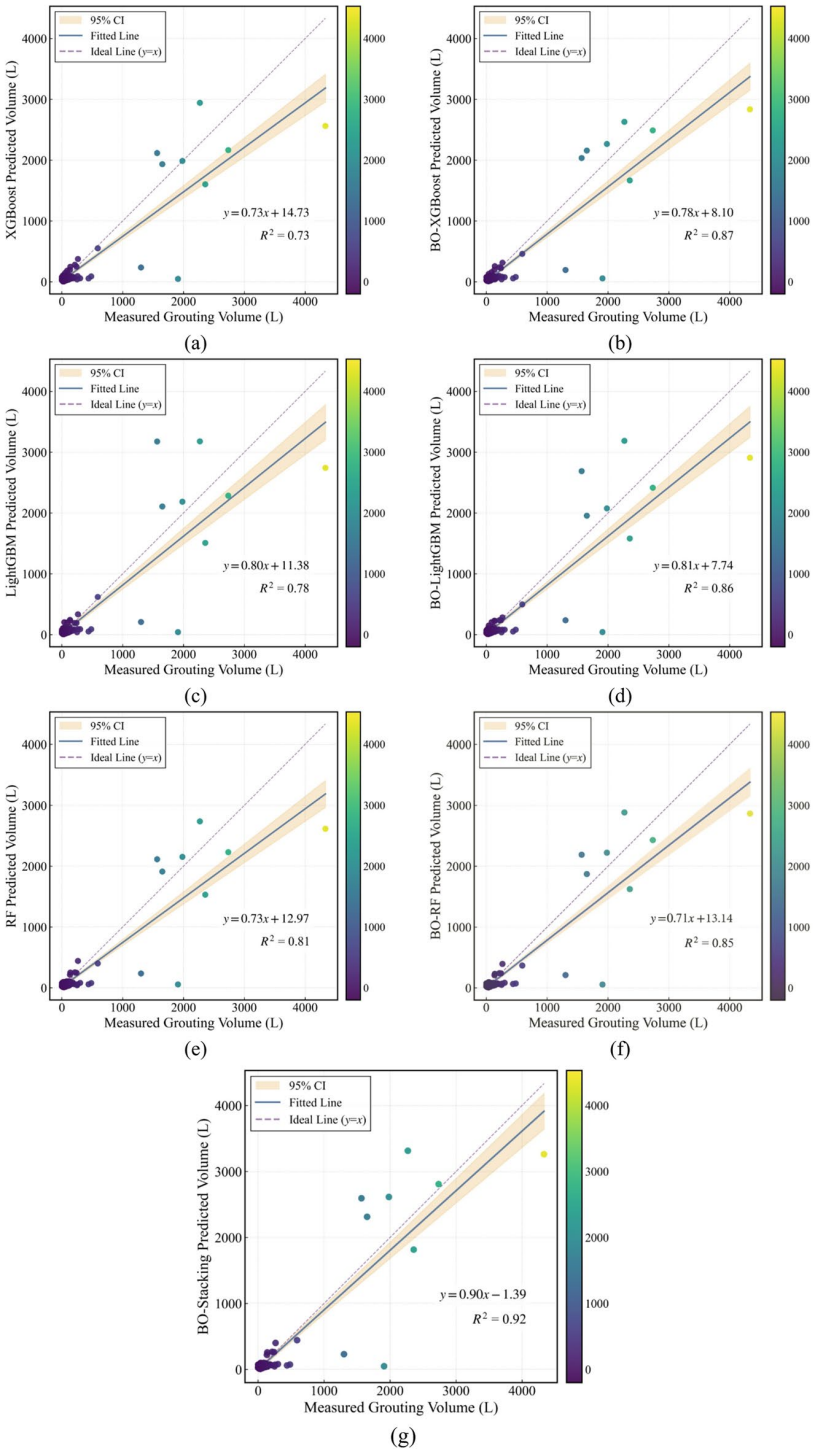
Scatter plots of predictive performance of the models on the test set: (**a**) XGBoost; (**b**) BO-XGBoost; (**c**) LightGBM; (**d**) BO-LightGBM; (**e**) RF; (**f**) BO-RF and (**g**) BO-Stacking.

To provide a sample-level assessment of predictive accuracy and systematic bias, Fig. 6 presents scatter plots of measured versus predicted grouting volumes for the seven models on the test set. Each point represents one test sample; the horizontal axis denotes the measured grouting volume, and the vertical axis denotes the corresponding model prediction. The purple dashed line represents the ideal line (*y* = *x*); the blue solid line indicates the fitted regression line; and the orange shaded band denotes the 95% confidence interval of the fitted regression line. This band reflects the uncertainty in the estimated trend between measured and predicted values.

As shown in Fig. 6, the baseline models without BO exhibited greater scatter dispersion. Especially in the medium-to-high grouting-volume range, many samples deviated substantially from the ideal line, indicating relatively large prediction errors in this range. The overall pattern also suggested the presence of potential systematic bias, along with pronounced deviations for some individual samples. Furthermore, the slope and intercept of the fitted regression line deviated noticeably from those of the ideal line. This suggested that the baseline models have an overall tendency toward systematic underestimation or overestimation of grouting volume changes.

Following BO optimization, the optimized models showed a clear improvement in scatter distribution. Most samples were more tightly clustered around the ideal line, with reduced dispersion and fewer apparent outliers. This suggested that the predictions were more consistent with the ideal agreement trend and that the fit across different grouting volume levels became more reasonable. Meanwhile, the fitted line was closer to the ideal line. This suggested that systematic bias was mitigated, and that the characterization of medium-to-high grouting-volume samples was improved.

Among all evaluated models, the BO-Stacking model achieved the best overall performance. Its sample points were closely concentrated near the ideal line, with a compact overall scatter pattern, indicating smaller deviations for most test samples. The fitted line was the closest to the ideal line, suggesting minimal systematic bias. Considering the compactness of the scatter distribution, the magnitude of sample-wise deviations, and the consistency between the fitted and ideal lines, the BO-Stacking model demonstrated favorable predictive accuracy and robustness on the test set. Consequently, it may be regarded as the best-performing model under the current evaluation setting.

### SHAP analysis

**Fig. 7:**
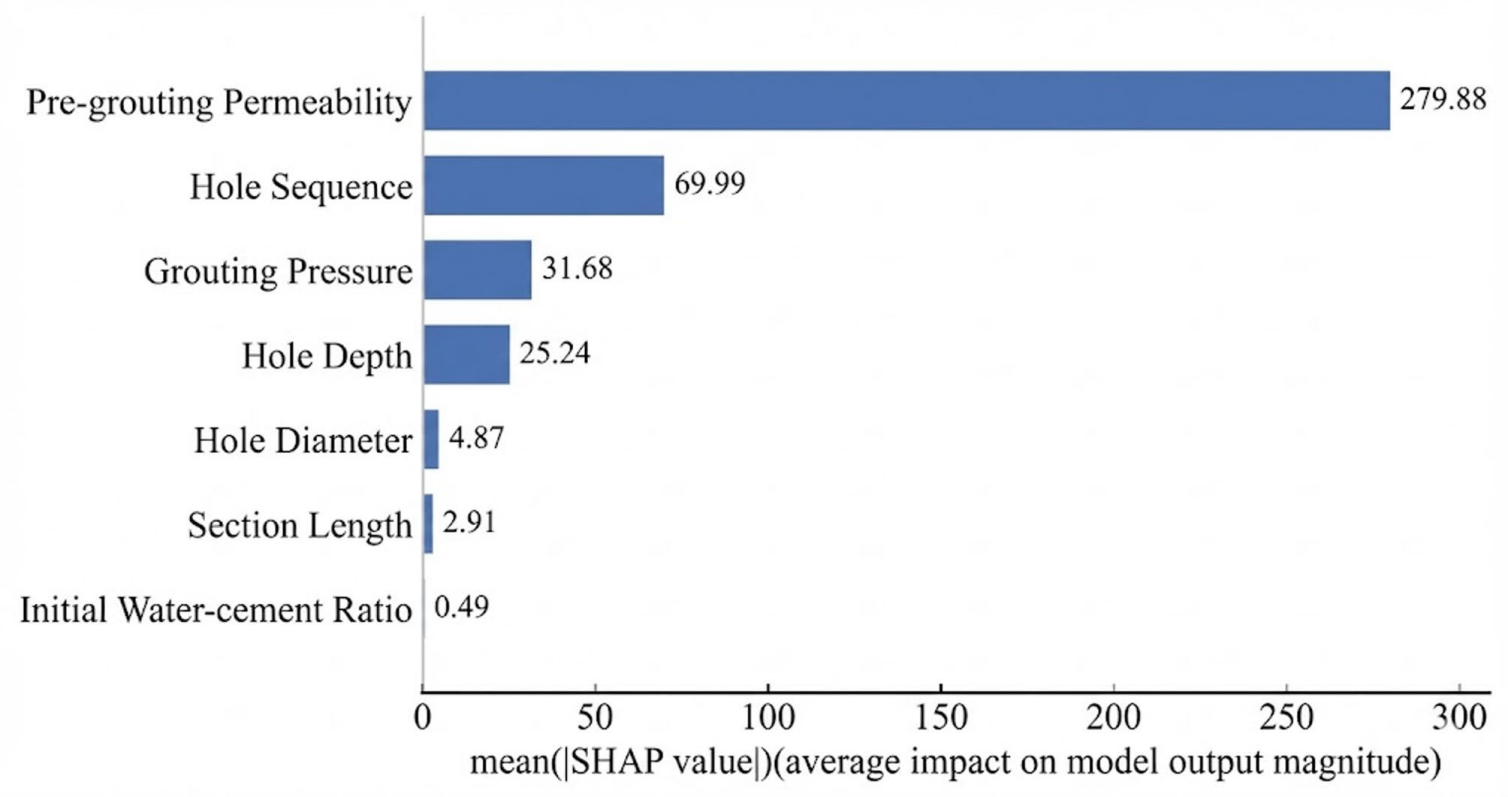
Variable importance ranking.

**Fig. 8:**
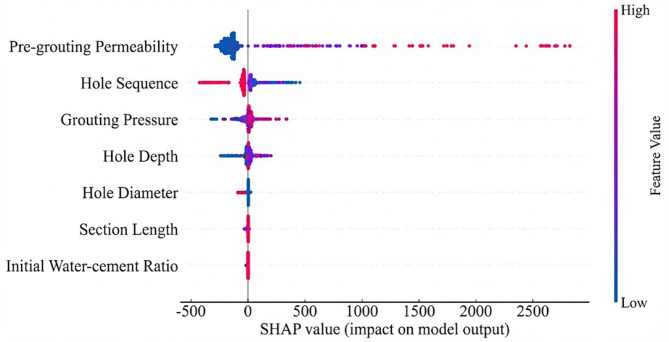
Feature SHAP values.

**Fig. 9:**
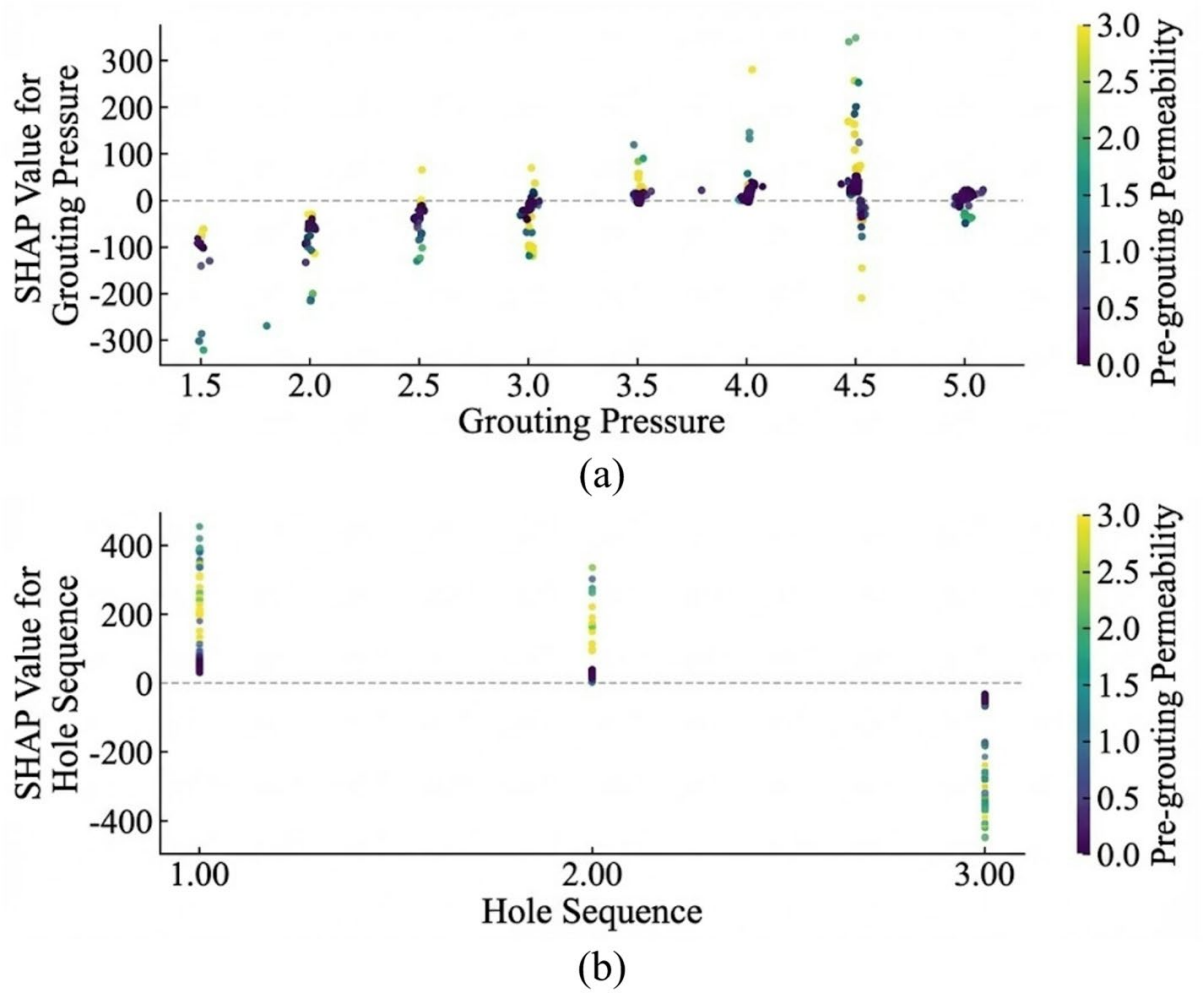
SHAP dependence plots: (**a**) pre-grouting permeability and grouting pressure; (**b**) pre-grouting permeability and hole sequence.

To address this interpretability limitation, SHAP analysis was applied to interpret the BO-Stacking model. SHAP quantified the marginal contribution of each input feature to grouting volume through an additive attribution framework. For clarity, all SHAP values reported in this section were expressed in liters (L). Figure 7 presents the global feature importance ranking based on mean absolute SHAP values, reflecting the overall contribution of each feature to grouting volume. Figure 8 further illustrates the direction and magnitude of the association between feature values and grouting volume across the feature value range. Figure 9 presents SHAP dependence plots to examine the conditional relationships between individual features and their attributed contributions to the predicted grouting volume.

As shown in Fig. 7, pre-grouting permeability had the largest mean absolute SHAP value of 279.88 L, substantially exceeding those of all other features. This finding was consistent with engineering knowledge, suggesting that pre-grouting permeability is the dominant feature associated with grouting volume under the current dataset. Among the construction parameters, hole sequence had the second largest mean absolute SHAP value of 69.99 L, suggesting that grouting sequence might notably influence stratum conditions. Grouting pressure and hole depth also exhibited moderate feature importance, with mean absolute SHAP values of 31.68 L and 25.24 L, respectively. By contrast, hole diameter, section length, and initial water–cement ratio had relatively small mean absolute SHAP values, indicating comparatively limited contributions to grouting volume under the current model and dataset.

As shown in Fig. 8, pre-grouting permeability showed a strong positive association with grouting volume. From the perspective of seepage mechanics, pre-grouting permeability may be regarded as a macroscopic indicator of fracture aperture and network interconnectivity. Samples with high permeability values were predominantly associated with positive SHAP values. This was consistent with the geological expectation that higher permeability may be associated with more developed fracture networks and correspondingly larger grouting volumes. Hole sequence showed a negative association with grouting volume in the SHAP analysis. As hole sequence number increased, the corresponding SHAP values tended to decrease, suggesting that grouting volume in later-sequence holes might be reduced following treatment of earlier holes. This pattern was broadly consistent with the principles of staged curtain grouting practice. Grouting pressure showed a positive association with grouting volume in the SHAP analysis. In grouting theory, cement slurry is generally characterized as a Bingham fluid with a finite yield stress. Grouting pressure acts as the driving force required to overcome this yield stress and drive slurry flow. Accordingly, higher grouting pressure may expand the diffusion radius of the slurry, potentially contributing to larger grouting volumes. This finding was broadly consistent with a previous grouting volume prediction study^42^, in which permeability-related variables were identified as the most influential features and process-related variables showed distinct contributions to model output.

Figure 9 presents SHAP dependence plots to examine the conditional relationships between selected engineering parameters and their attributed contributions to the predicted grouting volume. For grouting pressure, samples with higher permeability tended to exhibit larger positive SHAP values, suggesting that the contribution of grouting pressure to grouting volume might be more pronounced in highly transmissive strata. Similarly, the hole sequence dependence plot indicated that samples with higher permeability tended to exhibit larger SHAP magnitudes across sequence levels, suggesting that permeability might modulate the influence of hole sequence on grouting volume in transmissive strata. These findings were consistent with the view that grouting volume was influenced by multiple interacting factors rather than any single input variable.

## Discussion

### Comparative analysis with existing studies

Several previous studies have addressed grouting volume prediction using a range of machine learning and modeling approaches. Zhe et al.^42^ employed borehole image features combined with explainable machine learning methods, achieving an *R*^2^ of 0.848 for grouting volume prediction. In comparison, the BO-Stacking model proposed in the present study achieved better predictive performance under the current evaluation setting, with an *R*^2^ of 0.92. Although Tong et al.^22^ reported a higher *R*^2^ of 0.9675 for the ICEEMDAN-BO-GRU model, this result should be interpreted in the context of a different prediction setting. Their study focused on post-grouting prediction rather than on pre-grouting prediction. Variables such as pure irrigation time and end of injection rate may provide strong posterior information that improves model accuracy but are not available for advance prediction prior to grouting. Compared with the hybrid optimized stacking study by Zhu et al.^25^, the present study additionally introduced SHAP analysis to enhance model interpretability. Collectively, these studies were selected for comparison because they encompassed diverse modeling strategies, prediction settings, and levels of interpretability, thereby providing a representative benchmark for evaluating the proposed model.

### Research limitations

The data used in this study were collected from a single engineering project, in which the geological conditions, structural characteristics, and construction practices showed clear locality. Accordingly, the generalizability of the proposed model to other engineering contexts remains to be verified. It should be noted that this limitation is not unique to the present study, as grouting prediction studies have commonly relied on project-specific datasets from a single engineering project, such as the Dongzhuang hydraulic project dataset used by Sun et al.^24^ and the Huanggou Pumped Storage Power Station dataset used by Tong et al.^22^. Previous studies have suggested that grouting prediction may be improved by introducing more detailed fracture-related parameters^25^ or borehole image data^42^. Compared with those studies, the present study relied primarily on pre-grouting permeability as the main geological indicator. Additionally, the deep learning frameworks have been shown to improve grouting-related prediction in some contexts^20^, but the present study used relatively conventional machine learning algorithms. Furthermore, as this study was primarily motivated by practical engineering prediction rather than probabilistic statistical inference, it did not include statistical significance testing or systematic uncertainty quantification for the reported performance improvements.

## Conclusions

Based on field grouting data from a large-scale hydraulic project in Xinjiang, this study developed a BO-Stacking model for curtain grouting volume prediction and conducted a systematic evaluation of predictive accuracy, robustness, and interpretability. The main conclusions are as follows:

1. Bayesian optimization was applied to search complex hyperparameter spaces and identify improved configurations for the base learners. The BO-optimized XGBoost, LightGBM, and Random Forest models achieved better performance than their default baseline counterparts across all evaluated metrics. Compare to their default baseline counterparts, the BO-optimized XGBoost, LightGBM, and Random Forest models achieved consistent performance improvements across all evaluated metrics. BO provided a highly reliable and efficient approach for enhancing the predictive capabilities of base learners.
2. The BO-Stacking model demonstrated favorable predictive accuracy and robustness under complex geological conditions. Under the current evaluation setting, the model achieved the best performance across key metrics among all evaluated models, with an *R*^2^ of 0.92, an MAE of 70.19 L, and an RMSE of 187.07 L. In contrast to the benchmark models, which exhibited greater scatter dispersion and systematic bias in the medium-to-high grouting volume range, the predictions of the BO-Stacking model were more closely concentrated near the ideal line. The proposed model addressed certain limitations of conventional grouting volume prediction methods and provided robust methodological support for pre-grouting volume estimation under complex geological conditions, demonstrating significant engineering application value.
3. The SHAP analysis was broadly consistent with engineering knowledge. Pre-grouting permeability was the key feature associated with variation in grouting volume. Hole sequence was identified as the most influential construction parameter, while grouting pressure and hole depth also exhibited relatively important contributions to grouting volume. In contrast, hole diameter, section length, and initial water–cement ratio showed comparatively limited contributions to the predicted grouting volume. Grouting volume was influenced by multiple interacting factors spanning geological conditions, construction parameters, and slurry properties, rather than any single input variable. Overall, the SHAP analysis enhanced the interpretability of the BO-Stacking model and provided feature-contribution patterns broadly consistent with the engineering characteristics of curtain grouting.

## Data Availability

The data presented in this study are available on request to the corresponding author. The data are not publicly available due to privacy.
